# Twins! Microsatellite analysis of two embryos within one egg case in oviparous elasmobranchs

**DOI:** 10.1371/journal.pone.0224397

**Published:** 2019-12-02

**Authors:** Samantha A. Hook, Syafiq M. Musa, Daniel M. Ripley, Jean-Denis Hibbitt, Bianka Grunow, Timo Moritz, Holly A. Shiels

**Affiliations:** 1 Faculty of Biology, Medicine and Health, University of Manchester, Manchester, United Kingdom; 2 School of Earth and Environmental Sciences, University of Manchester, Manchester, United Kingdom; 3 School of Environmental and Natural Resource Sciences, Faculty of Science and Technology, Universiti Kebangsaan Malaysia, Selangor, Malaysia; 4 SEA LIFE Programmes and Engagement, SEA LIFE Weymouth, Weymouth, United Kingdom; 5 Leibniz-Institute of Farm Animal Biology, Dummerstorf, Germany; 6 Deutsches Meeresmuseum, Stralsund, Germany; 7 Institut für Zoologie und Evolutionsforschung, Friedrich-Schiller-Universität Jena, Germany; Consejo Nacional de Investigaciones Cientificas y Tecnicas (CONICET), ARGENTINA

## Abstract

Elasmobranchs display various reproductive modes, which have been key to their evolutionary success. In recent decades there has been a rise in the number of reported cases of foetal abnormalities including fertilised, double-embryos held within one egg capsule, hereafter referred to as twins. Previously, the occurrences of twin egg cases have been reported in two batoid and one shark species. We report the first cases of twins in three species of oviparous elasmobranchs: the undulate ray (*Raja undulata*), the nursehound (*Scyliorhinus stellaris*), and the small-spotted catshark (*Scyliorhinus canicula*). We investigated the genetic relationships between the twins in *S*. *stellaris*, and *S*. *canicula* using microsatellite markers. Whilst the *S*. *stellaris* twins displayed the same genotypes, we found that the *S*. *canicula* twin individuals arose through heteropaternal superfecundation. This is the first reported incidence of such a paternity in elasmobranchs. The relationship between environmental change and reproductive strategy in elasmobranchs is unclear and further research is needed to determine its effect on the prevalence and mechanisms of formation of elasmobranch twins.

## Introduction

Elasmobranchs comprise almost 1,200 species [1,2] of sharks and batoids (guitarfishes, sawfishes, skates and rays) [3] that display complex reproductive modes, characterised by low numbers of offspring that are born, or hatched, as active, fully-formed individuals [4]. All extant elasmobranchs employ internal fertilisation, with unique organ systems that increase the efficiency and likelihood of fertilisation, whilst minimising sperm wastage and the predation of unfertilised eggs [4,5]. Their diversity of reproductive traits is suggested to be a major selective advantage that has contributed to the group’s success [6]. However, these reproductive traits, coupled with slow growth, long gestation times, and late sexual maturity, have also increased the susceptibility of elasmobranchs to extinction in the current era of overexploitation and climate change [7].

Elasmobranchs display distinct reproductive modes: oviparity (egg-laying), and viviparity (yolk sac, histotrophic, oophagic, placental viviparity)[4,8]. Approximately 43% of elasmobranchs, predominantly the skates and benthic sharks, are oviparous [9,10]. The female reproductive organs of nearly all oviparous elasmobranchs consists of paired ovaries that each secrete oocytes into individual reproductive tracts (uteri) [11–14]. Each uterus comprises of oviducal (shelling) glands and muscular regions, before joining to form one lower uterus to release the fully formed eggs into the environment via the cloaca [11–14]. Typically, a single embryo is found within each egg case. Twin egg cases are rare, being reported in the oviparous skates *Leucoraja erinacea* [15], *Sympterygia bonapartii* [16] and one viviparious (yolk sac) shark species *Mustelus asterias* [17]. Double vitellogenic oocytes have also been observed in *Sympterygia acuta* [18]. Twin egg cases are only a common feature in the oviparious batoid species *Beringraja binoculata* [19], and *Beringraja pulchra* [20–23]. For this reason, Ishihara *et al*., (2012) proposed a new genus for these species, “Beringraja”[24].

Here we report two individuals formed in the same egg capsule in the undulate ray (*Raja undulata*), and fertilized double-embryo egg cases in the nursehound (*Scyliorhinus stellaris*) and the small-spotted catshark (*Scyliorhinus canicula*), here after referring to fraternal double-embryos as twins. Uniquely, our study employed microsatellite analysis to understand the reproductive origins of the double-embryos in *S*. *stellaris* and *S*. *canicula*.

## Methods

### Sample collection

On the 6^th^ of September 2013 an egg case containing two embryos from *R*. *undulata* was laid by a wild-caught mother, within a clutch of unknown size, at the SEA LIFE aquarium Weymouth, UK. The *S*. *stellaris* egg cases were laid in captivity by a wild-caught population at the Native Marine Centre (Portland, UK). The source population of *S*. *stellaris* individuals had either deposited eggs in captivity after copulation in the wild, or after copulation in the captive environment with other wild-caught individuals. The egg cases from *S*. *canicula* were from a captive breeding population held at the Deutsches Meeresmuseum (Stralsund, Germany), made up of a source population of both captive and wild individuals.

The egg cases from both shark species were sent to the University of Manchester, UK, at approximately 4 weeks, and 1 week, post-laying, for *S*. *canicula* and *S*. *stellaris*, respectively [25,26]. In Manchester, the embryos were held in 55L seawater tanks at 15˚C, dissolved oxygen > 95%, and 35ppt salinity, in a 12 hour light-dark cycle, until hatching. To ensure the nitrogenous waste contents was maintained at safe levels for the developing sharks, nitrate, nitrite and ammonia were routinely monitored, and water changes were carried out three times a week. The *S*. *stellaris* and *S*. *canicula* egg cases were photographed alongside a ruler, using a Canon PowerShot G16 camera, and the size of the egg cases, embryos, and external yolk sacs were measured using ImageJ [27]. The volumes of the external yolk sacs were calculated using the formula for an ellipsoid.

Ethical approval for work was granted from the Animal Welfare Ethical Review Board of the University of Manchester.

### DNA extraction, amplification and analysis

The *S*. *stellaris* and *S*. *canicula* embryos were fin-clipped post-hatch and the tissues stored in 98% ethanol for DNA extraction. A further 6 captive offspring *S*. *stellaris* samples were added to the dataset to investigate polymorphisms within the species. In *S*. *canicula*, the potential parents (fathers = 7, mothers = 11), 60 potential siblings, and the twin individuals from the captive breeding program were fin clipped to analyse parentage (in total n = 80). Samples were extracted using the Bioline Isolate II Genomic kit [28] with an extended digestion time of 10 minutes to maximise the genomic DNA yield. Genomic DNA (20-70ng/μl), was amplified with one primer cocktail containing 5mM of the three tail dyes (FAM, VIC and NED), 5mM of each forward microsatellite marker, and 10mM of each reverse microsatellite loci [29]. The 11 microsatellite primers and thermal cycling conditions were selected from Griffiths *et al*. [20]: Scan02, Scan03, Scan04, Scan05, Scan06, Scan09, Scan10, Scan12, Scan12, Scan15 and Scan16. PCR reactions consisted of 1μl of genomic DNA, 1μl of the primer cocktail, 3μl of ddH2O, and 5μl of QIAGEN Multiplex PCR Kit [30]. The products were genotyped using an ABI sequencer with GeneScan^™^ 500 LIZ^™^ dye Size Standard and scored using GeneMapper v.4.0 (Applied Biosystems). Allele scores were checked for user error in Microchecker [31].

GenePop (v 4.2) [32, 33] was used to calculate observed heterozygosity (*H*_o_), expected heterozygosity (*H*_*e*_) and number of alleles per locus (N_a_). Cervus [34] was used to calculate polymorphism information content (PIC) and frequency of null alleles F(Null) [29]. Parentage analysis for all offspring of *S*. *canicula* was determined using a full-likelihood and pair-likelihood-score combined (FPLS) method in Colony [35] and using a parent-pair log-likelihood ratio (LOD) analysis in Cervus [34]. Colony analysis was conducted under the assumption of female and male polygamy without inbreeding or clones. The simulation program within Cervus was used to produce 10,000 offspring and parental genotypes from allele frequencies taken from the North-Atlantic sampled by Gubili *et al*., [36] to generate statistically significant LOD scores at a strict confidence level of 95%. Microsatellite markers for both *S*. *stellaris* and *S*. *canicula* that displayed PIC values ≥0.500 were displayed for the twins and six individuals to visually highlight similarities and differences in the genotypes.

## Results

### Undulate ray, *Raja undulata*

**Fig 1:**
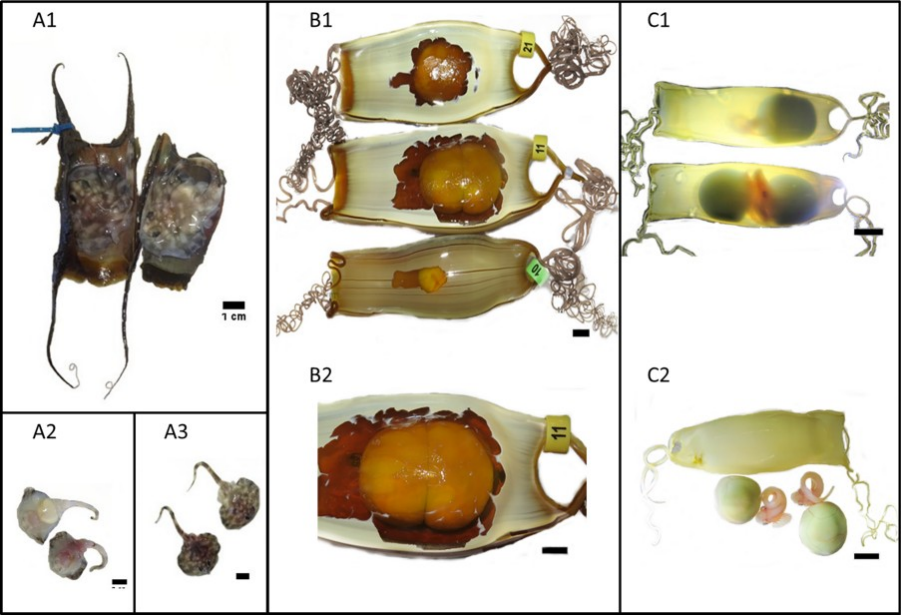
(A1) Two *R*. *undulata* juveniles in one, splayed open, egg case. (A2) The ventral surface and (A3) the dorsal surface of the two *R*. *undulata* twins. (B1) The *S*. *stellaris* twin egg case (middle) next to its paired-egg case and another egg (bottom). (B2) Closer view of the twined egg case. (C1) *S*. *canicula* twin embryos (bottom) next to a non-twin sibling (top). (C2) *S*. *canicula* twins after removal from their egg case. The black scale bars are 1cm in length.

The *R*. *undulata* twin embryo egg case length and width (excluding horns) was 58mm and 35mm respectively. While there was no reported difference at the time, these measurements show the egg case length to be slightly shorter when compared to other egg cases in the same clutch and those typical for the species (80.4 ± 4.4mm)[37–39]. During incubation the egg case was kept with others of the same clutch in 2500L natural seawater and maintained at 16.5˚C ± 1.8˚C with a dissolved oxygen of >95% and a salinity of 35ppt. Appropriate life support systems were also in place to ensure the nitrogenous waste contents were maintained at safe levels for the developing egg cases. On the 23^rd^ of April 2014 the egg case displayed signs of being unviable and so was opened, revealing two small dead juveniles (Fig 1A1). One juvenile was smaller and exhibited the early signs of decay with no evidence of a yolk sack while the larger juvenile was in the final stages of yolk sack absorption. It is unknown if the individuals were attached to a single yolk, or whether the egg consisted of two separate yolks. The disc width of the larger individual within the twin egg case was 4 cm (Fig 1A2 and 1A3), whereas a fully developed, healthy individual which hatched 8 days later from the same clutch had a disc width of 9 cm (not shown).

### Nursehound, *Scyliorhinus stellaris*

The *S*. *stellaris* twin egg case was larger than its paired egg case (i.e. the case laid at the same time as the twin egg case from the other oviduct, Fig 1B1). The twin egg case, excluding the tendrils, was 12.25 cm long, 5.65 cm wide and 3.70 cm deep. The average size of *S*. *stellaris* egg cases from the same cohort was 11.58 ± 0.1 cm, 4.32 ± 0.05 cm, 2.88 ± 0.06 cm in length (excluding the tendrils), width, and depth respectively (mean ± SEM, n = 12). At 12 weeks of development the external yolk sacs of the *S*. *stellaris* twins had a combined volume of 37.17 cm^3^ (Fig 1B2), more than twice that of a single yolk sac from a typical *S*. *stellaris* embryo reared under the same conditions (15.91 ± 0.93 cm^3^, mean ± SEM, n = 12). The twins survived for 12 weeks and developed to stage 20 defined by Ballard *et al*.[26], and stage 3 defined by Musa *et al*. [25], with total body lengths of 0.70 cm and 0.80 cm.

Genetic analysis of the *S*. *stellaris* twins revealed identical genotypic fingerprints on all 9 successfully amplified loci; individuals did not amplify with Scan06 and Scan16. The PIC was ≥0.500 for 4 of the 11 microsatellites for all samples (Table 1). As the individuals were developed from two yolks, rather than being monovular (i.e. two individuals with a single yolk), the monozygosity in the genotypes probably emanates from a lack of species-specific loci, and therefore a loss of interrogated diversity between the twins. Of the markers used Scan02, Scan09, Scan10, Scan13, and Scan15 displayed the highest *H*_o_ and *H*_*e*_ levels for the greatest number of individuals (87.5% to 100% of the sample size) and N_a_ for each was equal to or above 4 (Table 2). Overall, average genetic diversity for all eleven markers was *H*_o_ = 0.427 and *H*_*e*_ = 0.413 (Table 2).

**Table 1:** Genotypic information gathered from each microsatellite locus for the twin individuals (DY1, DY2) and a further randomly selected 6 possible siblings (SIB1-SIB6) of *S*. *stellaris* and *S*. *canicula* to display the genotype variance. Microsatellites with a polymorphism information content (PIC) value equal to or higher than 0.500 were used to display genotypes.

*S*. *stellaris*												
*ID*	*Scan02*			*Scan 09*			*Scan10*			*Scan15*		
**DY1**	135		137	131		133	268		276	250		252
**DY2**	135		137	131		133	268		276	250		252
**SIB1**	125		137	-		-	268		274	248		250
**SIB2**	125		139	133		133	274		274	248		250
**SIB3**	125		139	133		133	266		274	250		250
**SIB4**	137		139	133		137	268		268	248		250
**SIB5**	123		137	131		133	268		276	250		252
**SIB6**	133		141	129		131	274		276	258		260
***S*. *canicula***												
***ID***	***Scan02***		***Scan04***		***Scan06***		***Scan12***		***Scan15***		***Scan16***	
**DY1**	132	136	257	265	233	237	119	121	254	256	283	285
**DY2**	136	142	257	257	227	237	119	121	254	260	283	283
**SIB1**	132	134	257	267	233	237	119	121	254	256	283	285
**SIB2**	136	140	257	265	227	227	117	119	258	260	281	287
**SIB3**	132	144	257	265	227	233	119	121	256	260	279	281
**SIB4**	132	132	257	265	229	237	117	119	256	258	283	285
**SIB5**	132	132	265	265	227	229	117	119	254	258	283	285
**SIB6**	132	134	257	263	229	237	119	119	256	258	283	285

**Table 2:** Microsatellite information gathered from each locus for the entire population studied (including the twins) for *S*. *stellaris* and *S*. *canicula*. *N%* = Percentage of individuals scored, *Na* = numbers of alleles, *H*_*e*_ = expected heterozygosity, *H*_*O*_ = observed heterozygosity.

Loci information		*S*. *stellaris*				*S*. *canicula*			
Locus	Tail dye	*N%*	*Na*	*H*_*e*_	*H*_*o*_	*N%*	*Na*	*H*_*e*_	*H*_*o*_
Scan 02	NED	100	7	1.000	0.858	100	6	0.745	0.738
Scan 03	FAM	63	3	0.200	0.378	96	4	0.513	0.182
Scan 04	VIC	50	3	0.250	0.607	98	5	0.689	0.474
Scan 05	NED	100	2	0.125	0.125	100	4	0.543	0.450
Scan 06	FAM	38	1	0.000	0.000	100	9	0.730	0.713
Scan 09	VIC	88	4	0.714	0.626	100	2	0.025	0.000
Scan 10	NED	100	4	0.750	0.742	98	5	0.585	0.526
Scan 12	FAM	100	2	0.125	0.125	100	6	0.666	0.638
Scan 13	VIC	100	4	0.500	0.517	96	4	0.382	0.429
Scan 15	FAM	100	5	0.875	0.717	98	5	0.739	0.859
Scan 16	VIC	38	1	0.000	0.000	98	6	0.730	0.756

### Small-spotted catshark, *Scyliorhinus canicula*

The twin *S*. *canicula* egg case (Fig 1C1) was 6.52 cm in length (excluding the tendrils), 2.21 cm in width, and 1.57 cm in depth, making it slightly larger than the single embryo egg cases from the same clutch (6.33 ± 0.04 cm, 2.18 ± 0.05 cm, 1.36 ± 0.02 cm in length, width and depth respectively, mean ± SEM, n = 11). The lengths of the *S*. *canicula* twins at approximately 9 weeks post-laying were 4.67 cm and 4.69 cm, whilst the external yolk sac volumes measured 2.63 cm^3^ and 2.75 cm^3^. The lengths and key morphological features suggest that the twins reached somewhere between stages 28 and 32 of the Ballard *et al*. (1993) [26] developmental scale, and stage 4 of the Musa *et al*. (2018) [25] developmental scale. Due to concern for their well-being, the egg case containing the *S*. *canicula* twins was opened and the embryos (Fig 1C2) were transferred to individual artificial egg cases with larger dimensions and continued their development at 15˚C. Both animals survived with good health to hatch.

The average genetic diversity for all microsatellites was *H*_o_ = 0.524 and *H*_e_ = 0.577 (Table 3). The PIC was ≥0.500 on 6 of the 11 microsatellites (Table 1). Parentage analysis suggested that the twins derived from different paternities. Cervus parent pair non-exclusion probabilities all equal to or less than 1.30E-03 and Colony probability index of parent pairs were between 0.512 and 1.000 accurate (Table 2). Cervus gave more conclusive results in parentage due to the simulations for the log-likelihood ratio. These results suggest heteropaternal superfecundation (individuals from separate paternities, and therefore products of two distinct copulatory events) for the twins (DY1 and DY2) in *S*. *canicula* (Table 3).

**Table 3:** Results of parentage assignment from Cervus and Colony for *S*.*canicula*. Trio log-likelihood ratio (Trio LOD score) is the probability of relationship between the offspring, mother and father. *Probability Index* = probability of family clusters. DY = Twin individuals, SI = possible sibling individuals. * = no genotype matched.

	Cervus			Colony		
*Offspring ID*	*Candidate mother ID*	*Candidate father ID*	*Trio LOD score*	*Candidate Mother*	*Candidate Father*	*Probability Index*
DY1	MotherB6	FatherB1	7.92E+00	MotherB6	FatherB1	1
DY2	MotherB6	FatherB2	2.70E+00	MotherB6	FatherB2	0.512
SIB1	MotherB6	FatherB1	3.49E+00	MotherB6	FatherB1	0.512
SIB2	MotherB3	FatherB1	6.69E+00	MotherB3	FatherB1	1
SIB3	MotherB3	FatherB1	4.81E+00	MotherB3	FatherB1	1
SIB4	MotherB5	FatherB4	2.33E+00	MotherB2	*	1
SIB5	MotherB5	FatherB1	1.75E+00	MotherB2	*	1
SIB6	MotherB5	FatherB4	3.01E+00	MotherB2	*	1

## Discussion

Here we report the first incidence of an egg case containing two embryos in the oviparous *Raja* elasmobranch, the undulate ray (*R*. *undulata*). We also add two new species of oviparous benthic sharks (*S*. *stellaris and S*. *canicula*) to the list of elasmobranchii twin eggs, and provide the first genetic evidence of heteropaternal superfecundation in *S*. *canicula*.

The *S*. *canicula* and *S*. *stellaris* eggs all had two yolk sacs, indicating that two oocytes were released into the same oviducal gland for shelling in a single egg case. Genetic analysis revealed that the *S*. *canicula* twins were from heteropaternal superfecundation, meaning that each oocyte was fertilized by a different male, and thus suggesting sperm storage within the oviducal gland. Previous findings showed that females isolated from males for up to two years can produce fertile eggs [11], displaying longevity of the sperm and sperm storage which could account for the heteropaternal superfecundation reported here, if the female only mated with one individual during ovulation.

The mechanisms of double-embryo formation in the three oviparous elasmobranch species cannot be fully elucidated until development is tracked from ovary secretion, through the oviducal gland, to deposition. However, our findings are the first reported cases of shark twins in captive environments and provide the first evidence of heteropaternal superfecundation in a species of oviparous elasmobranch. The evolution of twin egg cases as a method of reproductive biology may have implications on the population persistence, if such individuals are unlikely to survive. However, if viable, increasing the number of individuals per reproductive output by producing twin egg cases would be advantageous. Overall there are an increasing number of reports on the occurrence of reproductive mutations such as double-embryo egg cases and conjoined individuals. The captive species which produce twin egg cases usually display high reproductive performance and plasticity [40], although without human input, twin egg cases typically do not succeed to hatch [18]. Considering the significant stress on wild populations of sharks and rays, further research is needed to understand and identify the mechanisms producing, and consequences of, elasmobranch twins.
